# Exploring the lived experiences of pregnant and breastfeeding teenage mothers in the conflict and post conflict Acholi sub Region, Uganda

**DOI:** 10.4314/ahs.v25i4.12

**Published:** 2025-12

**Authors:** Florence Ajok Odoch, Christopher Damulira, Kiyingi P Frank, Wandera O Robert, Adelline Twimukye

**Affiliations:** 1 Bugema University Kampala, School of post graduate studies, Department of Public Health; 2 Makerere University, Kampala, Department Counseling and Psychology; 3 Infectious Diseases Institute (IDI), Department of Counseling

**Keywords:** Teenage pregnancy and breastfeeding teenage mothers, post conflict sub Acholi region, LRA war

## Abstract

**Background:**

Globally, the ever-increasing numbers of teenage pregnancies reported is worrying, despite the commitment of the International Conference on Population and Development (ICPD) declaration, 1994. Behind these numbers, there are voluminous untold stories. This study documented vividly the lived experiences of teenage pregnant and breastfeeding mothers born during the Lord's Resistance Army LRA war in Acholi sub region that lasted for two decades (1986-2006).

**Methods:**

Qualitative cross-sectional case study design was used. Data was collected between January-March 2020. We interviewed 15 pregnant and lactating teenage mothers who were purposively selected. Data was collected using in-depth interviews (IDI) and analysed thematically using atlas ti version 5.

**Results:**

The lived experiences prior to their pregnancy, characterized by orphan-hood, school drop out, single parenthood, lack of basic needs and large family size posed server hardships and were detrimental. Equally, their experiences during and after pregnancy was troublesome as close to 80% of the participants were sole parenting. Stigma, worries, limited baby care skills and inadequate basic needs all mirrored very gloomy lived experiences of the teenage pregnant and breastfeeding mothers.

**Conclusion:**

The hardship life experienced prior to the teenage pregnancy was so detrimental and as such greatly contributed to the teenage pregnancy and motherhood. These factors were largely caused by the two decades of the LRA war that weakened the family and societal fabrics. The study recommends promotion of sexuality education, mentorship and skills building for young parents (mothers).

## Introduction

Teenage pregnancy and teenage motherhood is defined as a teenage girl, usually within the ages of 13-19, becoming pregnant and giving birth^1^,^2^,^3^. Teenage pregnancies can bring about major health and social problems with unique medical and psychosocial consequences for the teenagers and society at large^4^. Despite the ICPD of 1994, globally, teenage pregnancy remains a major health and development challenge, it's considered the leading cause of newborn and maternal mortality in developing countries^5^,^6^,^7^. Pregnancies among teenagers are associated with several adverse health, educational, social and economic outcomes^8^,^9^. And yet, every year, the world continues to record increasing numbers of teen pregnancies, with over 21 million teen pregnancies in 2020 up from 16 million in 2016 & up from 12 million in 2014^10^,^11^. Exacerbated by conflicts, wars and violence, children, adolescents and worse still, adolescent girls who become mothers; experience triple-edged challenges that come with bodily changes (growth and development) and the added parental responsibility in a society where social fabrics and family protection system have been weakened by war and conflict as it was the case of the Lord Resistance Army (LRA) war in Northern Uganda^12^. According to the Women's Refugee Commission and Save the Children, United Nations High Commission for Refugees (UNHCR) & United Nations Population Fund (UNFPA)^13^, in any war and conflict settings, reproductive health risks for adolescents become greater. These risks are attributed to forced sex, increased risk-taking and reduced availability of and sensitivity to ASRH services. Girl children become more vulnerable to sexual abuse and exploitation which in turn increases their vulnerability to sexually transmitted infections including HIV and AIDS, unwanted pregnancies and unsafe abortion^14^.

Northern Uganda recorded some outstanding negative indicators on ASRH including; teenage pregnancy of 23.6% compared to 19.2%, unsafe abortion of 70% compared to 54%. Adolescent start at child bearing was 30.3% as compared to 24.9% the national average respectively^15^,^16^. These outstanding ASRH figures could be due to the conflict and post conflict effects. This study investigated the lived experiences of teenage pregnant and breastfeeding mothers in a bid to explore and document their lived experiences before, during and after giving birth, the fact that the LRA war had weakened the family and societal protective fabrics.

The negative health consequences experienced by adolescents can pass from one generation to the next. And in this case, children born by mothers who are adolescents may experience the same and the cycle of teenage motherhood continues. Clearly documenting the lived experiences of teenage pregnant and breastfeeding mothers in conflict and post conflict era will greatly contribute to understanding ways of promoting and mitigating children safety and protection in conflict settings.

Theory of psychosocial development by Erik Erikson 1902-1994^17^ postulates that development occurs throughout one's lifespan through social and emotional interaction with the environment. These interactions help children develop skills to handle social conflicts and crisis through the eight stages to overcome everyday social conflict and crisis. These include trust vs. mistrust, autonomy vs. shame & doubt, initiative vs. guilt, industry vs. inferiority, industry vs. confusion, intimacy vs. isolation. Therefore, given the challenges that come with adolescent growth, development and pregnancy, this theory guided this study to explore the lived experiences of the teenage pregnant and breastfeeding mothers in conflict and post conflict setting.

## Methods

### Study design

In January-March 2020, we conducted a qualitative cross-sectional case study design^18^ that employed phenomenological approach^19^,^20^ to collect empirical data from 15 teenage pregnant and breastfeeding mothers aged 10-19 years, who were receiving antenatal care (ANC) and postnatal care (PNA) from selected health facilities in Gulu, Kitgum and Pader districts of Acholi sub region, Northern Uganda. The study targeted teenage pregnant and breastfeeding mothers who had babies less than 1 year at the time of the study. Using exploratory and descriptive methods, the case study documented the lived experiences of the participants before, during and after giving birth. Using the health facility as an entry point, all the level of health care facilities that offer ANC and PNC were listed and purposively picked.

### Setting

The study was carried in the districts of Gulu, Kitgum and Pader. The region affected by over two decades of the LRA war which led to massive destruction of lives and property, family life was greatly interrupted, and people were displaced into Internally Displaced Persons (IDP) camps^21^. The estimated population of the three districts is 854,397 people; Gulu having 436,345, Kitgum with 240,048 and Pader with 178,004 and with an estimated 23.2% of young people aged 10-19 years in the three districts translating in to a total of 198,220 adolescents^22^ and these were born during and after the LRA war. The adolescent and teenage pregnancy rate in the region stood at 23.6%, and those who have had a live birth stood at 19.1%. 23.

### Sampling

The study^18^ targeted teenage pregnant and breastfeeding mothers who were receiving ANC and PNC from selected health facilities in Gulu, Kitgum and Pader were selected. From each district, three (03) health facilities were purposively selected based on the level of facility; one H/C III, one H/C IV and 1 district hospital). In total, nine health facilities were selected. Using the ANC/PNC registers, two (02) participants were purposively selected; each selected participant was scheduled for an interview during their next upcoming ANC/PNC appointment visit. The midwife at the health facility scheduled their visits and requested the participants to come with a guardian. Prior to the interviews, consent was obtained from the participants. These were emancipated minors.

### Data Collection

Data was collected using in-depth interviews (IDI). For purposes of ethical consideration, all the necessary information was provided to the participants for consenting and assenting. One-on-one interviews were conducted in separate nurses' rooms, this ensured privacy. Data was collected from 15 participants on all the aspects of the research question based on saturation approach^24^. Research assistants who were trained in qualitative data handling, collected data in Acholi language using IDI guides developed to answer the research questions. The IDI guides were piloted and revised majorly on choices of words which were not uniformly understood. Data was audio-recorded and complemented with written notes. The IDI guides were developed with broad open-ended questions to encourage detailed discussions of the study topic. The study answered the following questions: What were the life experiences of the participants' before, during and after giving birth? The duration of the interviews lasted on average 60 minutes per participant.

### Data analysis

The audio recordings were transcribed verbatim and expanded notes generated from the filed notes were used. Atlas ti version 5 computer software was used to facilitate data searching, sorting and then copying it into separate files. Based on the codes developed, the data were analyzed and reported using themes and quotes. Based on summaries of major themes picked, tallies and counts were converted in to numerical numbers and percentages depicting the socio-demographic information of the participants.

## Results

Summary of the participants' socio-demographics and households characteristics

**Table 01:** Socio-demographic characteristics and household characteristics of the participants

T/Ms codes (P 1-P15)	Age of T/Ms	Level of education for T/Ms	Orphan status	Economic activities prior to pregnancy	Household size	Guardian & r//ship with T/Ms	Occupation of guardian (P/F)
P1	14	DRP LP	½ orphan	Schooling	7	Mother(sm)	p/sf
P2	14	DRP LP	total orphan	Domestic work	4	Aunty	p/sf
P3	14	DRP LP	alive but (sp)	Paid domestic work	6	Mother(sm)	p/sf
P4	15	DRP LP	total orphan	Paid domestic work	6	Husband	p/sf
P5	15	Secondary	½ orphan	Schooling	7	Mother (sm)	p/sf
P6	15	DRP LP	both alive	Schooling	5	Husband	p/sf
P7	16	DRP LP	total orphan	Paid domestic work	7	Grandmother (sm)	p/sf
P8	16	DRP LP	total orphan	Paid domestic work	7	Sister	Hair dresser
P9	17	DRP LP	both alive	Schooling	6	Husband	p/sf
P10	17	DRP LP	alive but (sp)	Paid domestic work	6	Mother (sm)	p/sf
P11	16	DRP LP	total orphan	Domestic work	8	Husband	p/sf
P12	17	DRP LP	½ orphan	Domestic work	9	Mother(sm)	p/sf
P13	17	DRP LP	total orphan	Paid domestic work	12	Grandmother (sm)	p/sf
P14	17	DRP LP	½ orphan	Domestic work	5	Mother(sm)	p/sf
P15	18	DRP LP	½ orphan	Paid domestic work	7	Mother(sm)	p/sf

Key: P- Participants; DRP LP = Dropped at lower primary; T/Ms= Teenage Mothers; N/A =Not applicable, r/ship-relationship, p/sf=peasant/subsistence farmer, sp= step parenthood, sm= single mother

### Factors affecting pregnant and breastfeeding teenage mothers

Various individual, family, household, and community and factors were shown to generally influence decisions around pregnant and breastfeeding teenage mothers in in the conflict and post conflict Acholi sub region.

**Table 02:** Pregnancy and breastfeeding experiences of the teenage mothers

Level of experiences	Themes
Personal and individual	▪ Education level and School dropout, ▪ Orphan hood status, ▪ Economic and livelihood challenges ▪ Limited knowledge and skills to care for the baby ▪ Worries about their looks and what will happen ▪ Sole parenting and lack of basic needs.
Family, household	▪ Household size ▪ guardianship of the teenage mothers ▪ household source of livelihood
Community	▪ Stigma and discrimination ▪ Categories of men who impregnated the teenage mothers

### Individual level experiences

At a personal or individual level, education level and school dropout, orphan hood status, economic and livelihood challenges, limited knowledge and skills to care and handle the baby, stress and worries, sole and single parenting and lack of basic needs to support themselves were evidently reported.

### Education level and School drop out

Seventy three percent (73%) of the participants had dropped out of school prior to their pregnancy and 27% dropped out due to teen pregnancy. Fourteen (14/15) participants stopped in lower primary. Participants attributed dropping out of school as the main cause for their pregnancy as they had nothing meaningful to occupy them as depicted:

“I was already out of school and I did not have much to do …, I then looked for a job as a housemaid…during my work time, I ended up getting a man …” Individual interview, 14 years old teen mother from Pajule H/C IV, Pader District.

Being out of school for girls has greater and far reaching impact including the risk of teenage pregnancy and early marriage. Mothers with low education attainment carry greater risks in terms of poor health seeking behaviors, negative attitudes towards education and low future aspiration for the children.

### Orphan hood status

The majority (80%) of the teen mothers were orphans who had lost one or both parents. Paternal orphan-hood stood at 75%. The experiences of being orphaned prior to teen pregnancy were detrimental as depicted in some of the quotes.

“My father died, my mother has been struggling alone to provide the basics for us…, life was so difficult, I dropped out of school and got married” individual interview, 17 years old teenage mother Kilak H/C III, Pader District. Orphan hood has far reaching impact on the life of children, parents' death comes with lots of psychological, emotional and economic implications to the children; most often struggles to meet the basic needs of life, risk dropping out of school, involvement crimes, drugs addiction, violence and teenage pregnancy.

Economic and livelihood activities that the teenage mothers were engaged in prior to pregnancy eleven (11) (73%) out of the 15 participants were engaged in domestic activities to support them and their families as depicted.

“My mother could not give me money to buy personal requirements; life was not easy at home, so I had to look for a job in order to get some money…” Individual interview, 18 years old teenage mother, Kitgum General Hospital, Kitgum District.

The economic hardships experienced by the teenage mothers may carry long term impact on the life of the mothers and the children, most often the vicious cycle of households' poverty and economic deprivation will move from generation to generation due to their inability to obtain the basic factors for economic development such as education.

Limited knowledge and skills to care for and handle the baby, worries about their appearances and looks as the pregnancy grew and the stomach growing bigger, sole and single parenting and lack of basic needs to support themselves. Some quotes were picked as depicted below:

### Limited knowledge and skills to care for the baby

Some participants reported limited knowledge and skills to handle and care for the baby which was a major challenge as depicted.

“I do not have adequate knowledge and skills to handle and take care of a baby, and am worried of what will happen to me after giving birth …” individual interview a 14 year old Pader H/C III, Pader District.

“I feared that I may sleep on my baby at night and wondered if I sleep on the same mattress with my baby; won't I sleep on the baby?” Individual interview, a 15 years old, teenage mother, Cwero H/C III, Gulu District

### Worries about their looks and what will happen

“I needed to look good, but my mother has not bought for me any maternity dress, I fear moving in public places like markets so I stay home mostly…” individual interview, a 15 years old, teenage mother, Cwero H/C III, Gulu District.

“The health workers told me that I was still young and may not give birth normally. They advised me to go to the main hospital, this worries me …” (individual interview, a 14 years old teenage mother Omiyanyima H/C III, Kitgum District).

The implication for this limitation/challenge creates anxiety, and it is not healthy for both the mother and baby in terms of the risk of her getting preterm birth and or low birth weight. And in a setting where there no classes/training to prepare mothers for their first and new-born, hands-on training and mentorship could help mitigate the anxiety. Teenage mothers could be mentored by older and experienced mothers, who could be their own moms or midwives.

### Sole parenting and lack of basic needs

Over 70% of the participants reported that they were living under the guardianship of other people rather than the responsible men and as such had the sole responsibility with so many challenges as depicted.

“I have many challenges, if my baby is sick I get so worried especially at night, I stay-up alone since my grandmother is now old …” individual interview, a 15 years old teenage mother, Lalogi H/C IV, Gulu District.

“I don't have money to buy requirements for my delivery, the man who impregnated me disappeared, am so worried …” individual interview, a 16 years old teenage mother, Pader H/C III, Pader District.

I stay home with my mother and do house chores and yet I have many needs that my mother can't afford, may be if I was leaving with my boyfriend, probably he could buy them for me…” --individual interview, a 16 years old teenage mother, Pader H/C III, Pader District.

Mothers who experience lots of stress and emotional torture while pregnant risk passing on certain traits and mental health problems to their unborn such may include, low brain development of the baby (Down syndrome) mental retardation and imbalances their growth and development. To mitigate the disastrous effects that such stress, parents/guardians of such young mothers needs to have the capacity to support and provide the basics for the mother and baby.

### Households and family

#### The guardianship of the teenage mothers

Majority twelve (12/15) teenage mothers were living under the guardianship of women who were either their own mothers, grandmothers, an aunty or a sister. And of these (9/12) guardians were living as single mothers.

#### The household source of livelihood

All the guardians were peasant farmers and farming was the main source of livelihood reported. Farming was majorly for domestic consumption and as reported prior, 47% of the teenage mothers sought paid domestic work to able them earn an income to buy personal requirements including Vaseline, pads etc.

“sometimes as a girl, there are things I needed such as Vaseline, pads, but my mother was not in position to provide, because she was struggling to look for money” individual interview, 17 years old teenage mother, Pader H/C III, Pader District.

#### Household size

The households had a relatively big number of dependents/or siblings. On average, from the 15 participants, seven (7) siblings/dependents per household.

“We were 7 children in total, my father died, life was difficult, my mother each day struggled to get the basic needs we needed at home…” Individual interview, 16 years old teenage mother, Omiyanyima H/C III, Kitgum District. The conditions in the households revealed that the families struggled to get the basics of life, compounded by the traumatic experiences; the effects of stress on teenage pregnancy are enormous with detrimental impact on their pregnancies.

#### Family and community experience

Stigma and discrimination at family and community level. The participants all mentioned stigma and discrimination as one of the issues, especially blames from their brothers and fathers and generally from the community. These were quotes depicted;

“My father and brother wanted me to be married off, that I was a disgrace and bringing shame to the family” Individual interview, 14 years old teenage mother, Omiyanyima H/C III, Kitgum District).

“I experienced a lot of community stigma, many people talked about my pregnancy and wanted me married off, only my mother stood by me…” Individual interview, a 15 years old teenage mother, Cwero H/C III, Gulu District.

“I feared so much to line up during antenatal care (ANC) with other older women who most often were fond of backbiting…” Individual interview, a 14 years old teenage mother, Lalogi H/C IV Gulu District.

The sources of stress of the teenage mothers were multiform; the teenage mothers were at the mercy of God. Given the implication of stress on pregnancies, families and communities need to be supported to develop positive attitudes, look at a bigger picture of life of the teenage mothers beyond the pregnancy.

### Categories of men who impregnated the teenage mothers

The participants were asked to talk about the man who was responsible for their pregnancy.

**Table 03:** The category of men who impregnated the teenage mothers

a) Category of men who impregnated to teenage mothers	# of men	%age
Men who were out of school	111	73.3
Men who were in school (another school)	01	6.7
Men in same school (a school mate)	03	20
**Total**	**15**	**100**
b) **Current guardianship and taking responsibility for the pregnancy**		
Men who denied responsibility	08	53.3
Men who accepted responsibility	04	26.7
Parents refusal to let the teenage mother go to the man responsible (child to child sex)	03	20
**Total**	**15**	**100**

### Pregnancy by a school mate

Four (04/15) teenage mothers were still schooling at the time they conceived. Three (3/4) (75%) had been impregnated by a school mate and the boys were all in upper primary. This was child to child sex that ended up into teenage pregnancy. One participant had this to say…

“I had sex with my school mate, after some time, I started falling sick, after visiting the hospital, the nurse told me, I was pregnant, I then dropped out of school and life became so difficult” Individual interview, 14 years, Cwero H/C III, Gulu District.

The implication of these findings indicates, children are beginning to experience early sexual debut, schools should therefore integrate sexuality education as part of learning in schools and are equipped with skills to protect themselves from teenage pregnancies.

### Denial of pregnancy by the men who impregnated the teenage mothers

Eleven (11/15) teenage mothers were impregnated by men from the community. Of these, eight (8/11) (73%) men denied the pregnancy as depicted below. The denial and/or disappearance by the men responsible were due to; fear of being arrested for defilement and lack of resources to start a family.

“I was so sickly … when I went to the hospital, the health worker informed me that I was pregnant and asked me to come with the man who impregnated me … when I told him he refused and then disappeared, I have not seen him since then, am told he went to Kampala …” Individual interview, 14 years old teenage mother from Pajule H/C IV, Pader District.

“When I told my boyfriend that I was pregnant he denied and threatened to kill me if I ever disclose …” Individual interview, 15 years old teenage mother Lalogi HCIV, Gulu District.

The implication of these findings reveals that the child protection law needs to be reinforced both at the family level and in the community in order to bring the perpetuators to face the law and pay for their crimes.

## Discussion

This study explored the lived experiences of teenage pregnant and breastfeeding mothers in conflict and post conflict Acholi sub region in Northern Uganda. Majorly, the discussions focus on the lived experiences of the participants, before, during and after delivery. It was revealed, that; the hardship life experienced prior to them becoming pregnant in terms of individual socio-demographics and household characteristics including education level and school dropout were (73%), being orphan (80%), economic hardship (93% peasantry farming) engaging in domestic or causal paid work (50%), the household composition such as single-mother headed households (60%) and large house size were the major risk factors to teenage pregnancy. These findings are similar with a couple of previous studies^25^,^26^ which indicate low economic background and limited access to educational services; other scholars report educational disruptions and financial difficulties as major risk factors to teenage pregnancy^27^,^28^. In line with the household characteristics, household composition factors such as single headed households were reported to negatively impact family life^29^,^30^.

The categorization of men who were responsible for the teenage pregnancies; twenty percent (20%), reported child to child sex which happened at school among the learners (upper primary), this is something new that this study revealed, previous studies have majorly reported on teachers- learners sexual relationships^31^,^32^,^33^ in which educators' sexual misconduct (ESM), was reported and that about 39% of the students exposed to sexual misconducts were at risk of getting pregnant and could drop out of school.

In regards to denial of pregnancy and sole parenting by teenage mothers reported at 73%, various factors including, fear of being arrested for defilement, relocation and lack of resources to start a family were reported; these findings are in line with studies done in Eastern Cape Province of South Africa^34^,^35^. The sole parenting; stigma, worries, difficulties in meeting basic needs for the mother and child, inadequate knowledge and skills of baby care were the major challenges experienced by the teenage mothers. Similarly, in Kavango regions, Namibia^36^, indicates that teenage mothers face stigma, worries, lack of knowledge and skills to parenting. In the same direction^37^, reports that worries, stress, regret, guilt, and stigma from society members, along with limited financial support and lack of knowledge, advice, and emotional support before, during, and after pregnancy were major problems for teenage mothers. Further more, in the Southern Hho-hho region of Swaziland, they reported that parents, partners, peer groups, health personnel, teachers, church leaders, and communities fail to empower the teenage mothers with the necessary knowledge and skills and this most often leads to poor outcomes for both the mother and baby^38^,^39^.

## Conclusions

The hardship life experiences lived prior to the teenage pregnancy was so detrimental and were as such responsible for their teenage pregnancy. These factors were largely caused by the over two decades of the Lord's Resistance Army (LRA) war that led to a total breakdown in family and societal fabrics. Further, the denial of parental responsibility by the men who impregnated them, stigma, worries, lack of basic needs, inadequateor lack of life skills and parenting skills by the teenage pregnant and breastfeeding mothers all mirrored very gloomy lived experiences of the teenage pregnant and breastfeeding mothers.

## Recommendations

### Areas for further study

Do a qualitative study to explore the family and community support to teenage pregnant and breastfeeding mothers

Carry out a qualitative study on the impact of the LRA war on the family and community support to teenagers and adolescents in regards to sexual and reproductive health and rights.

### Areas for practice and policy

Promote life skills and sexuality education for children both in school and out of school (children in upper primary). This should be guided by policy and implementation framework.

Teenage mothers need to be equipped with basic parenting skills; old mothers, aunties, grandmothers and health workers (midwives and nurses) can mentor/train teenage mothers through demonstration and illustration showing them the “what” and “how” of parenting.

## List of abbreviations

AIDS: Acquired immune deficiency syndrome
ASRH: Adolescent Sexual Reproductive Health
ANC: Antenatal care H/C Health Centres
HIV: Human immune Virus
ICPD: International Conference on Population and Development
IDI: In-Depth Interviews
LRA: Lord's Resistance Army
PNC: Post-natal care
STIs: Sexually Transmitted Infections Acknowledgements

## Responsibility of Authors

The corresponding author carried out the research and drafted the manuscript.

The other authors had equal input in to the review process and approval of the final paper.
